# Magnetic Resonance-Guided Laser-Induced Thermal Therapy for Recurrent Brain Metastases in the Motor Strip After Stereotactic Radiosurgery

**DOI:** 10.7759/cureus.919

**Published:** 2016-12-07

**Authors:** Aditya Iyer, Casey H Halpern, Gerald A Grant, Sayantan Deb, Gordon H Li

**Affiliations:** 1 Department of Neurosurgery, Stanford University School of Medicine; 2 Medical School, Stanford University School of Medicine

**Keywords:** laser thermography, brain metastases, thermocoagulation, thermotherapy, motor cortex

## Abstract

The authors report a challenging case of a brain metastasis located in the motor cortex, which was not responsive to radiosurgery. Use of a novel technique, magnetic resonance-guided laser-induced thermotherapy (MRgLITT), resulted in the complete obliteration of the lesion without adverse effects or evidence of tumor recurrence at follow-up. This case illustrates that MRgLITT may provide a viable alternative for patients with brain metastases refractory to radiosurgery or in deep locations, where both stereotactic radiosurgery (SRS) and surgical resection may be ineffective.

## Introduction

The management of brain metastases located in eloquent regions of the brain remains a clinical challenge. Mainstay treatment options include stereotactic radiosurgery, microsurgical resection, whole-brain radiation, chemotherapy, or a combination of these strategies. Postoperative motor function after resection and stereotactic radiosurgery (SRS) is highly variable and often poor. Additionally, there are few salvage options for symptomatic tumor recurrence after stereotactic radiosurgery. Magnetic resonance-guided laser-induced thermotherapy (MRgLITT) has become an increasingly widespread modality for ablation of both primary brain tumors as well as metastatic disease due to its minimally invasive technique as well as its selective ability to minimize damage to surrounding tissue. This is a specialized approach for careful frameless stereotactic placement of a laser fiber into the lesion, followed by laser thermal ablation of the tissue in the MRI suite. As heat changes the gradient echo MR signal, the change in tissue temperature can be observed in real time as MR images are obtained. When the pathologic tissue is satisfactorily heated and inactivated, the procedure is stopped.

There are currently no reported outcomes for patients who have undergone MRgLITT for brain metastasis in the eloquent regions of the brain, particularly in the motor strip. In this report, we present a patient who successfully underwent MRgLITT for a solitary brain metastasis in the motor strip who failed initial SRS and had an excellent neurological outcome.

## Case presentation

**Figure 1:**
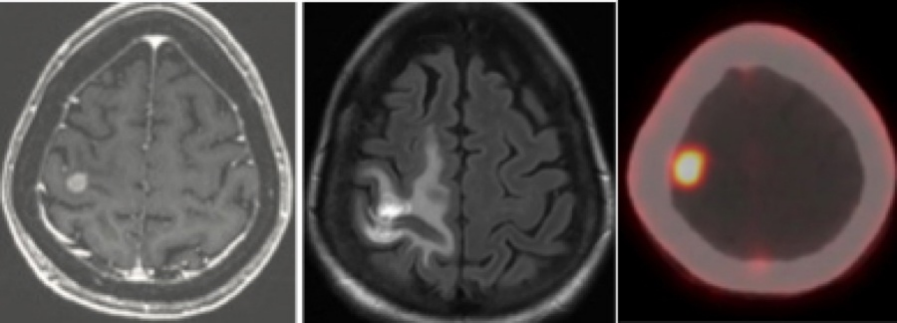
Pre-radiosurgery Images Pre-radiosurgery T1 post-gadolinium MRI demonstrating a 1 cm metastasis in the right motor strip, adjacent to the right-hand motor area (left). At six-month follow-up, there was significant post-radiosurgery fluid-attenuated inversion recovery (FLAIR) signal (middle), which demonstrated significant fludeoxyglucose (FDG) uptake on PET-CT (right).

The patient is a 65-year-old woman with a history of non-small cell lung cancer with known metastases to the brain. She had undergone whole-brain radiation with subsequent CyberKnife treatment of a 3 cm residual right anterior frontal and right central sulcus metastases. Follow-up imaging at six months demonstrated increased size of the treated right central sulcus lesion. An 18F-fluoropropylglutamate (18F-FSPG) PET demonstrated an area of increased uptake in the right central sulcus consistent with tumor progression (Figure 1). Informed patient consent was obtained for her treatment.

She additionally developed intractable motor seizures consisting of contralateral arm and leg twitching as well as left arm and hand weakness. Given the tumor progression despite radiosurgery as well as the high risk of paralysis following open surgical resection, it was decided to use MRgLITT performed with the Visualase® system (Medtronic Inc, Minneapolis, MN) as a salvage treatment option. 

### Technique

**Figure 2:**
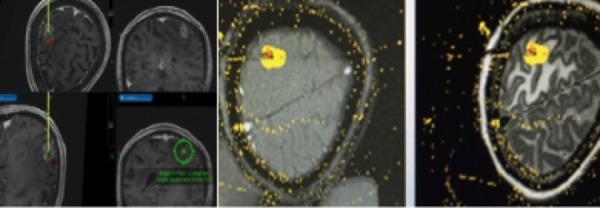
Stealth Plan Stealth plan for insertion of the Visualase laser into the lesion (left). The final ablation result is seen on the T1 and fluid-attenuated inversion recovery (FLAIR) axial MRI scans, which demonstrate minimal thermal damage to surrounding tissue (right).

The patient was taken to the operating room and had six skull fiducials placed. She then underwent registration with the O-arm® (Medtronic Inc, Minneapolis, MN), which was merged to the CT scan and prior MRI scan intraoperatively. The patient was then positioned in prone in Mayfield® pins. Using our previously described frameless approach [1], we registered the skull fiducials and used the StealthStation® Navigation (Medtronic Inc, Minneapolis, MN) to guide an entry point with an error of 0.3 mm. Using the Vertek® biopsy tool (Medtronic Inc, Minneapolis, MN), we then placed the biopsy needle into the lesion and confirmed that the lesion was indeed recurrent non-small cell lung cancer metastasis and not radiation necrosis. The laser fiber was then placed down the planned track and an intraoperative CT scan was taken with the O-arm to confirm proper placement and that no hemorrhage was caused. We merged this O-arm CT with her preoperative CT scan and MRI. The patient was then taken down to the MRI suite. The target appeared excellent, and we performed a test dose heating in the region of the tumor anteriorly with excellent results. We then heated the tumor bed to 70 degrees for three minutes. Four burns total were fashioned after we pulled the catheter back. The ultimate length of the laser ablation was about 2.2 cm along the axis of the tumor (Figure 2). There were no radiographic complications at the end of the procedure. The patient returned to the recovery room in stable condition.

**Figure 3:**
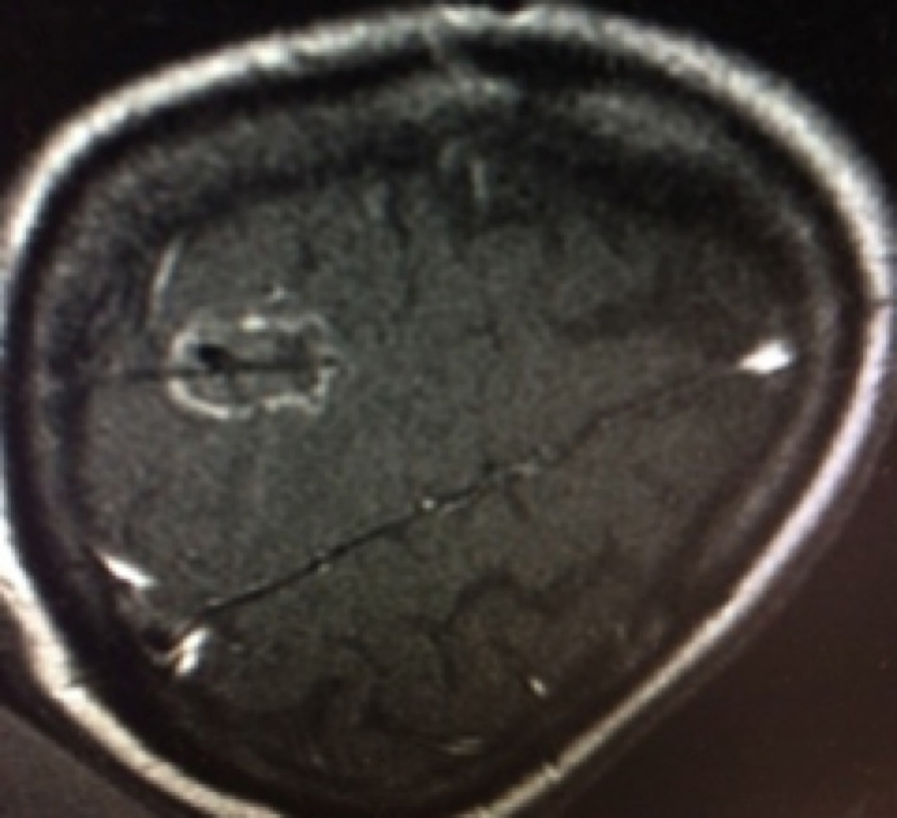
Six-Month Follow-up Scan Postoperative axial T1 post-gadolinium MRI scan at the six-month follow-up demonstrates evidence of the original tract into the lesion, with no new areas of enhancement concerning for tumor recurrence.

Postoperatively, the patient regained full motor function and there was no evidence of tumor recurrence or seizure activity at the six-month follow-up (Figure 3).  

## Discussion

An estimated 9% - 17% of cancer patients will develop brain metastases during the course of their illness [2]. The prevalence of multimodality management has increased the number of longer-term survivors while preserving functional outcomes. However, the management of lesions of the motor cortex has remained a clinical challenge.

Clinicians will often advocate for surgical resection of large tumors with significant surrounding vasogenic edema. Obermuller, et al. reported a series of 56 patients who underwent microsurgical resection of metastases in the motor cortex, 12 (21%) of whom who experienced worsening paresis postoperatively [3]. Similarly, Walter, et al. reported a series of 20 patients who underwent surgery in or adjacent to the motor cortex with a 15% incidence of postoperative paresis [4]. However, Kellogg, et al. reported 17 patients who underwent resection of metastases within the motor strip with symptoms stabilizing or improving in 94% of patients at the three-month follow-up [5]. These series indicate that postoperative outcomes can be highly variable with surgical resection.

Stereotactic radiosurgery is another treatment option in the initial management of patients with brain metastasis in the eloquent cortex. Radiosurgery has a reported rate of symptomatic neurological complications ranging from 6% - 10%, the most common complication of SRS being the development of radiation necrosis, occurring in approximately 24% of lesions treated [6]. The largest series of brain metastasis in the motor cortex treated with stereotactic radiosurgery alone consisted of 96 patients, of whom only 31% had improvement in motor function, with 50% of patients remaining stable and 19% worsening. Tumors greater than 9 cm^3^ had significantly worse outcomes with SRS alone [7].

MRgLITT is a new therapeutic option for patients with brain metastases, and large series are needed to better understand its utility; early studies are promising. On principle, MRgLITT therapy offers coagulation of tissue with sharp margins, which reduces surrounding tissue edema and may be an important adjuvant or alternative to both surgery and SRS. Several preliminary studies using MRgLITT have been promising [8].

Carpentier, et al. reported six patients with small (< 2.5 cm) brain metastases that had failed radiosurgery. There were no reported complications or radiographic evidence of post-procedural edema [9]. Torres-Reveron, et al. [10] reported six patients who underwent MRgLITT for brain metastases throughout the cerebrum, of whom only one had tumor progression at three months and required salvage surgical resection. There were no reported complications. Regarding metastases in eloquent regions, Hawasli, et al. reported a case of a patient with 3.3 cm colorectal adenocarcinoma metastasis in the insular cortex successfully treated with MRgLITT alone [11].

## Conclusions

MRgLITT may provide a viable alternative for patients with brain metastases refractory to radiosurgery or in deep locations, where both SRS and surgical resection may be ineffective. Larger series are needed to validate the utility of this promising new modality in the management of brain metastases. 
